# An Optimized LIME Scheme for Medical Low Light Level Image Enhancement

**DOI:** 10.1155/2022/9613936

**Published:** 2022-09-22

**Authors:** Yue Kun, Gong Chunqing, Gao Yuehui

**Affiliations:** Tianjin Modern Vocational Technology College, Tianjin, China

## Abstract

The role of medical image technology in the medical field is becoming more and more obvious. Doctors can use medical image technology to more accurately understand the patient's condition and make accurate judgments and diagnosis and treatment in order to make a large number of medical blurred images clear and easy to identify. Inspired by the human vision system (HVS), we propose a simple and effective method of low-light image enhancement. In the proposed method, first a sampler is used to get the optimal exposure ratio for the camera response model. Then, a generator is used to synthesize dual-exposure images that are well exposed in the regions where the original image is underexposed. Next, the enhanced image is processed by using a part of low-light image enhancement via the illumination map estimation (LIME) algorithm, and the weight matrix of the two images will be determined when fusing. After that, the combiner is used to get the synthesized image with all pixels well exposed, and finally, a postprocessing part is added to make the output image perform better. In the postprocessing part, the best gray range of the image is adjusted, and the image is denoised and recomposed by using the block machine 3-dimensional (BM3D) model. Experiment results show that the proposed method can enhance low-light images with less visual information distortions when compared with those of several recent effective methods. When it is applied in the field of medical images, it is convenient for medical workers to accurately grasp the details and characteristics of medical images and help medical workers analyze and judge the images more conveniently.

## 1. Introduction

**Figure 1:**
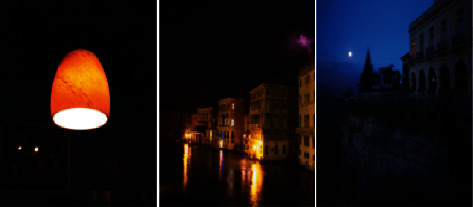
Naturally captured low-light images.

Low-light images are a ubiquitous image model. Its main features are low brightness and a darker area at the center of the image. Such images have low visibility, are not easy to observe and analyze, and are not conducive to related applications, which makes digital image processing face major challenges.  Figure 1 provides three such examples, such as the painting on the wall in the first example, almost “buried” in the dark. In order to enhance low-light images, people have researched many classic algorithms, but the processing results of these algorithms are more or less problematic. For example, the enhancement algorithm based on wavelet transform [1] can not only describe the outline of the image but also highlight the details of the image due to its multiresolution characteristics, but it has little effect on the change of the image contrast. The histogram-based enhancement algorithm [2] mainly uses histogram equalization to improve the contrast of the image, but the grayscale of the processed image is reduced, and some details are lost. In [3], the authors try to enhance the contrast while maintaining the naturalness of the lighting. While it prevents the output image from being overenhanced, its performance is not very appealing in terms of efficiency and visual effects. The illu-adj algorithm proposed in [4] is able to process the *H* and *I* components separately and achieve image enhancement in the HSI color space. However, the image processed by this scheme is still dark, and the effect is not good. In conclusion, so far there is no perfect method for low-light image enhancement.

Inspired by the human visual system (HVS) [5], this paper proposes a simple and effective innovative method: a low-light image enhancement method based on an optimized LIME scheme. This paper compares this innovative algorithm with four other algorithms: multilevel color consistency theory (MSRCR) [6], simultaneous reflection and illumination estimation algorithm (SRIE) [7], light map estimation-based method (LIME) [8], and the multibias fusion method (MF) [9]. Experimental data show that our proposed method has unique advantages in luminance order error (LOE), visual information fidelity (VIF), and subjective vision.

Furthermore, the main contributions of this paper can be summarized as follows:

1. This paper proposes a low-light image enhancement scheme that optimizes LIME.
2. We find the optimal exposure ratio so that the composite image has a good exposure effect in the underexposed area of the original image.
3. We design a low-complexity calculation scheme to obtain the weight matrix.
4. In order to improve the performance of the output image, we increase the postprocessing part. In the postprocessing part, the optimal grayscale range of the image is adjusted, and the image is denoised and reconstructed using the BM3D model.

## 2. Optimize LIME Scheme

**Figure 2:**
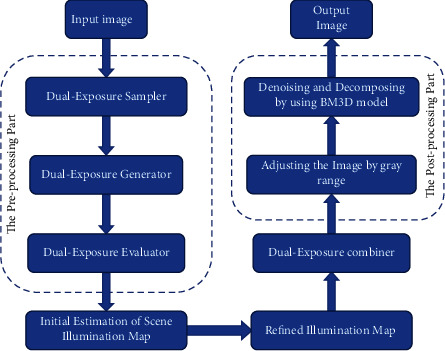
Algorithm framework.

Our optimized LIME scheme consists of three parts: preprocessing, weight matrix construction by the LIME method, and postprocessing. The overall algorithm framework is shown in Figure 2.

**Figure 3:**
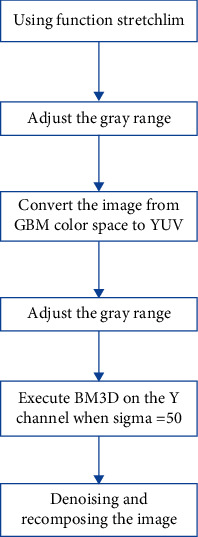
Flowchart of the postprocessing algorithm.

Among them, the first part consists of a double-exposure sampler, a generator, and an evaluator. The sampler can obtain better exposure of the camera response model; the generator can synthesize the image at the optimal exposure rate to make it in the underexposed area of the original image. The inner exposure is good; since the weight matrix of all pixels is nonuniform, i. e., well exposed pixels are given larger weights, and poorly exposed pixels are given smaller weights; therefore, the weights of all images are weighted using the estimator matrix, which is evaluated such that the output matrix is normalized per pixel. Second, we use the LIME algorithm to determine the weight matrix when the two images are fused. By fusing the composite image and the input image according to this weight matrix, an enhanced image with all pixels well exposed can be obtained. Finally, the postprocessing part improves the performance of the output image by adjusting the optimal grayscale of the image (the flowchart of the postprocessing algorithm is shown in Figure 3). In addition, we use the BM3D model to denoise and reconstruct the image to further optimize the performance after image enhancement.

According to our algorithm framework, the enhanced image can be defined as

(1) $$\begin{array}{c} R^{c}=W\circ P^{c}+\circ g\left(P^{c},\hat{k}\right)\text{.} \end{array}$$

Among them, *c* is the index of the three color channels, *R* is the result of image enhancement, *W* represents the weight matrix, *g* is the brightness transfer function (BTF), which $\hat{k}$ represents the optimal exposure rate, and *P* is the input original image,∘ which represents the correspondence between the two matrices The elements of the position are multiplied. It is obvious from this formula that to get an enhanced image, we can start with 3 parts: double exposure evaluator (evaluate *W*), double exposure generator (determine *g*), and double exposure sample (determine $\hat{k}$).

### 2.1. Double Exposure Sampler

The double exposure sampler will determine the optimal exposure $\hat{k}$ for the resulting image so that the input image and the resulting image represent as much information as possible, while leaving the composite image well exposed in areas of the original image that were underexposed.

A well-exposed image has better visibility than an underexposed or overexposed image and can give people more information. So, we have to find the best exposure $\hat{k}$ to provide the most information. To find $\hat{k}$, we define image entropy as

(2) $$\begin{array}{c} H\left(B\right)=-\overset{N}{\underset{i=1}{\sum }}p_{i}\cdot \log_{2}p_{i}\text{,} \end{array}$$

where *B* represents the luminance component of the image and *p*_*i*_ represents the ith element of the histogram of *B*.

Since the entropy of a well-exposed image is higher than that of an underexposed or overexposed image, therefore, it is reasonable to use entropy to find the optimal exposure. We compute the optimal exposure $\hat{k}$ by maximizing the image entropy that enhances brightness, which is expressed as

(3) $$\begin{array}{c} \hat{k}=argmaxH\left(g\left(B,k\right)\right)\text{.} \end{array}$$

Since the image entropy first increases and then decreases with the increase of the exposure rate $\hat{k}$,$\hat{k}$ can be solved by the one-dimensional minimization method. To improve computational efficiency, we resize the input image to 50 × 50 during optimization.

### 2.2. Double Exposure Generator

In this part, we use the camera response model to implement the double exposure generator, and the camera is constructed by BTF *g*.

To estimate *g*, we select two images with different exposures, named *P*_0_ and *P*_1_, respectively. The BTF model can be described by a two-parameter function as

(4) $$\begin{array}{c} P_{1}=\left(g\left(P_{0},k\right)\right)=\beta P_{0}^{\gamma }\text{.} \end{array}$$

Among them, *k* is the exposure rate, and *β* and *γ* are the parameters related to the exposure rate *k* in the BTF model. It is observed that the different color channels have approximately the same model parameters. The fundamental reason is that for a typical camera, the response curves of different color channels are approximately the same.

Since the BTF of most cameras is nonlinear, our BTF *g* can usually be solved by

(5) $$\begin{array}{c} g\left(P,k\right)=e^{b\left(1-k^{a}\right)}P\left(k^{a}\right)\text{,} \end{array}$$

where *β*and *γ* are two model parameters, which can be calculated from camera parameters a, *b* and exposure *k*. We assume that no information about the camera is provided, and we need to use a fixed camera parameter (*a* = −0.3293, *b* = 1.1258) that can fit most cameras.

### 2.3. Double Exposure Evaluator

The design of the weight matrix *W* is the key to realizing the enhancement algorithm, which can enhance the low contrast in the underexposed areas while preserving the contrast in the well-exposed areas. We need to assign larger weight values to well-exposed pixels and smaller weight values to underexposed pixels. Intuitively, the weight matrix is positively related to the scene illumination. Since areas of high illumination are more likely to be well exposed, they should be assigned large weight values to preserve their contrast. Inspired by the LIME method [10], this paper calculates the weight matrix by the following formula:

(6) $$\begin{array}{c} \begin{array}{c} W_{h}\left(x\right)\leftarrow \underset{y\in \Omega \left(x\right)}{\sum }\frac{G_{\delta }\left(x,y\right)}{\left|\sum_{y\in \Omega \left(x\right)}G_{\sigma }\left(x,y\right)\nabla_{h}\hat{T}\left(y\right)\right|+\epsilon }\\ W_{v}\left(x\right)\leftarrow \underset{y\in \Omega \left(x\right)}{\sum }\frac{G_{\delta }\left(x,y\right)}{\left|\sum_{y\in \Omega \left(x\right)}G_{\sigma }\left(x,y\right)\nabla_{v}\hat{T}\left(y\right)\right|+\epsilon } \end{array}\text{.} \end{array}$$

Here, ∇_*h*_ represents the gradient in the horizontal direction,∇_*v*_ represents the gradient in the vertical direction, $\hat{T}$represents the initial estimate of the light map,*ϵ* is a very small constant to avoid a denominator of 0, Ω(x)is the area centered on the pixel *x*, and *y* is the position index within the area. G_*δ*_(x, y)can be expressed as

(7) $$\begin{array}{c} G_{\sigma }\left(x,y\right)\propto \exp\;\left(-\frac{di\;st\left(x,y\right)}{2\sigma^{2}}\right)\text{,} \end{array}$$

where di st(*x*, *y*)is used to measure the spatial Euclidean distance between the *x* and *y* positions.

### 2.4. Light Map Estimation

As one of the earliest color constancy methods, the Max-RGB [11] method attempts to estimate the illuminance by finding the maximum value of the three color channels (*R*, *G*, and *B* channels), but this estimation can only improve the global illuminance. In this paper, in order to deal with nonuniform lighting, we choose to use the following initial estimates:

(8) $$\begin{array}{c} \hat{T}\left(x\right)\leftarrow \max_{c\in \left\{R,G,B\right\}}P^{c}\left(x\right)\text{.} \end{array}$$

For each individual pixel *x*, the rationale behind the above operation is that the illuminance is at least the maximum value of the three channels at some pixel location.

For the initial estimation of light maps, a good solution should preserve both overall structure and smooth texture details. To solve this problem, we propose to solve the following optimization problems:

(9) $$\begin{array}{c} \min_{T}{\left\|\hat{T}-T\right\|}_{F}^{2}+\alpha {\left\|W\circ \nabla T\right\|}_{1}\text{,} \end{array}$$

where *α* is a coefficient that balances these two terms, ‖·_*F*_‖ and ‖·_1_‖ represent the *F*-norm and 1-norm, respectively,**T** is the original light map. ∇T is the first derivative filter, and it contains ∇_*h*_**T**(horizontal) and ∇_*v*_**T** (vertical). In Equation (9), the first term guarantees fidelity between $\hat{T}$ and **T**, while the second term considers (structure-aware) smoothness.

### 2.5. Low Complexity Solution

On carefully analyzing the problem (9), it is not difficult to find that the origin of the iterative process is the sparse weighted gradient term, that is, the 1-norm term||**W**∘∇**T**||_1_. The combination of the 1-norm and the gradient operation of *T* makes the calculation somewhat complicated. Therefore, in order to reduce the computational complexity, formula (10) is obtained:

(10) $$\begin{array}{c} \lim_{\epsilon ⟶0^{+}}\underset{x}{\sum }\underset{d\in \left\{h,v\right\}}{\sum }\frac{W_{d}\left(x\right){\left(\nabla_{d}T\left(x\right)\right)}^{2}}{\left|\nabla_{d}T\left(x\right)+\epsilon \right|}=W\circ \nabla T_{1}. \end{array}$$

That is,**W**∘∇**T**_1_ can be approximated by using ∑_*x*_∑_*d*∈{*h*, *v*}_**W**_*d*_(*x*)(∇_*d*_**T**(*x*))^2^/|∇_*d*_**T**(*x*)+*ϵ*|. Therefore, the problem in (9) can be approximately described as

(11) $$\begin{array}{c} \min_{T}{\left\|\hat{T}-T\right\|}_{F}^{2}+\alpha \underset{x}{\sum }\underset{d\in \left\{h,v\right\}}{\sum }\frac{W_{d}\left(x\right){\left(\nabla_{d}T\left(x\right)\right)}^{2}}{\left|\nabla_{d}\hat{T}\left(x\right)\right|+\epsilon }. \end{array}$$

Since equation (11) contains a quadratic term, the problem can be solved directly by solving the following equation:

(12) $$\begin{array}{c} \left(I+\underset{d\in \left\{u,v\right\}}{\sum }D_{d}^{T}Diag\left({\tilde{W}}_{d}\right)\left(D_{d}\right)=-t\right)\text{,} \end{array}$$

where ${\tilde{w}}_{d}$ is the vectorization of ${\tilde{W}}_{d}$, which is expressed as${\tilde{W}}_{d}\left(x\right)\leftarrow W_{d}\left(x\right)/\left|\nabla \hat{T}\left(x\right)\right|+\epsilon$. Diag(**x**) is a diagonal matrix constructed with the vector *x*. is a symmetric positive definite Laplacian matrix, and there are many practical solutions in [12–16]. After doing all the above steps, we can combine them to get an enhanced image *R*^*c*^.

### 2.6. Adjust the Grayscale Range

In this section, we use the imadjust function in MATLAB to adjust the grayscale range of the image and set the gamma value in the function to 0.9 to achieve better visual effects.

### 2.7. Denoising and Restructuring Using BM3D Models

To further improve the visual effect, we also employ a denoising recombination technique. Considering the comprehensive performance, this paper chooses the BM3D [17] model.

BM3D (block matching 3D, a three-dimensional block matching algorithm) can be said to be one of the best denoising algorithms at present. The main idea of this algorithm is to find similar blocks in the image for filtering processing, which can be divided into two steps: one is basic estimation, and a simple denoising operation is performed. The second is the final estimation, and more detailed denoising is performed to further improve the peak signal-to-noise ratio.

In this paper, to further reduce the amount of computation, we convert the input image P from the RGB color space to the YUV color space and perform BM3D only on the *Y* channel. In addition, the noise level is different for different input regions due to different magnifications. BM3D models have the same effect on different areas. Therefore, to avoid imbalances in processing, such as some locations (dark areas) being well removed and some locations (bright areas) being oversmoothed, we use the following operations to maintain the balance.

(13) $$\begin{array}{c} R_{f}\leftarrow R\circ T+R_{d}\circ \left(1-T\right)\text{,} \end{array}$$

where **R**_*d*_ and **R**_*f*_ are the results after denoising and recombination.

## 3. Result Analysis

**Figure 4:**
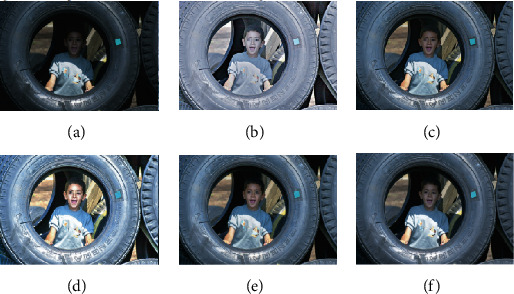
BOY1 enhancement results. (a) Original. (b) MSRCR. (c) SRIE. (d) LIME. (e) MF. (f) Proposed.

**Figure 5:**
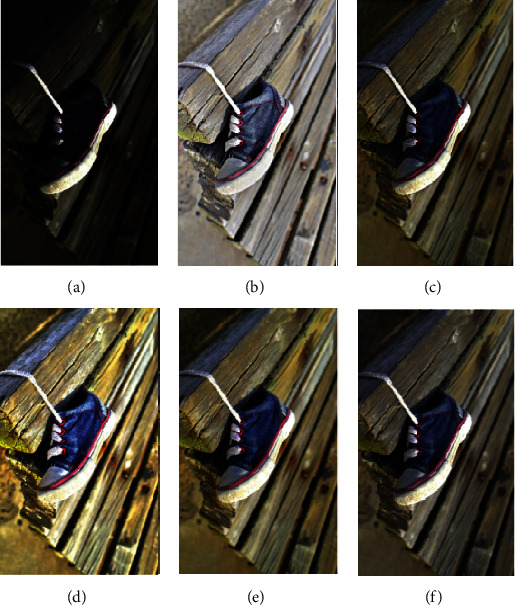
SHOE enhancement results. (a) Original. (b) MSRCR. (c) SRIE. (d) LIME. (e) MF. (f) Proposed.

**Figure 6:**
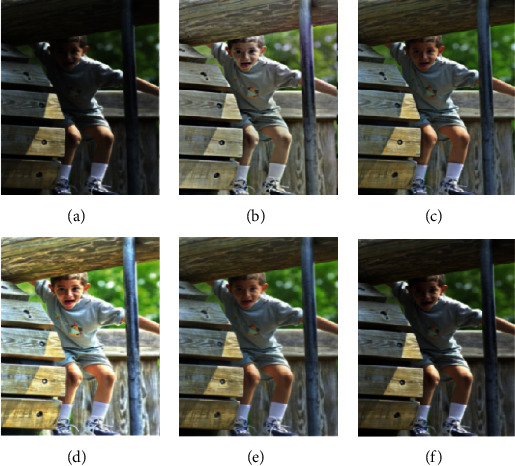
BOY2 enhancement results. (a) Original. (b) MSRCR. (c) SRIE. (d) LIME. (e) MF. (f) Proposed.

**Figure 7:**
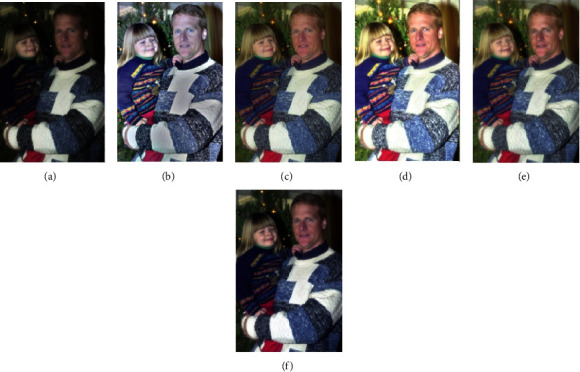
FATHER enhancement results. (a) Original. (b) MSRCR. (c) SRIE. (d) LIME. (e) MF. (f) Proposed.

**Figure 8:**
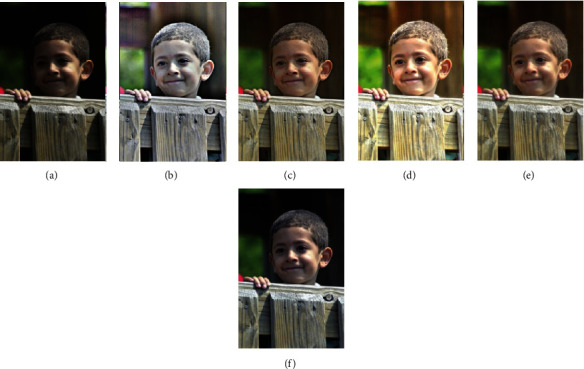
BOY3 enhancement results. (a) Original. (b) MSRCR. (c) SRIE. (d) LIME. (e) MF. (f) Proposed.

In this section, we compare our scheme with other methods, including MSRCR, SRIE, LIME, and MF methods. The results are shown in Figures 4–8.

To verify the effectiveness of the proposed algorithm, we process some typical low-light images. The names are BOY1, SHOE, BOY2, FATHER, and BOY3. Here are their comparison results:

Furthermore, to demonstrate the effectiveness of the proposed algorithm, we evaluate the enhanced images using the following 3 metrics:

1. Lightness order error (LOE)
2. Visual information fidelity (VIF)
3. Human subjective visual evaluation

### 3.1. Luminance Sequence Error (LOE)

The relative order of brightness represents the light source direction and brightness changes, and the naturalness of the enhanced image is related to the relative order of brightness in different areas. Therefore, we adopt the luminance order error (LOE) as an objective measure of performance. The definition of LOE is as follows:

(14) $$\begin{array}{c} LOE=\frac{1}{m}\overset{m}{\underset{x=1}{\sum }}\overset{m}{\underset{y=1}{\sum }}\left(U\left(Q\left(x\right),Q\left(y\right)\right)⊕U\left(Q_{r}\left(x\right),Q_{r}\left(y\right)\right)\right)\text{,} \end{array}$$

where *m* is the number of pixels. The function *U* (*p, q*) outputs 1 where *p* ≥ *q* and 0 otherwise. The symbol “⊕” represents the exclusive or operator. **Q**(*x*) and **Q**_*r*_(*x*) are the maximum values between the *R*, *G,* and *B* channels at the *x* position of the enhanced image and the reference image, respectively. The lower the LOE, the better the image enhancement and the more natural brightness can be maintained. The results are shown in Table 1.

### 3.2. Visual Information Fidelity (VIF)

VIF models the quality assessment problem as an information fidelity criterion, quantifying the mutual information between the reference image and the distorted image relative to the image information extracted by HVS. The VIF metric [18] can be expressed as

(15) $$\begin{array}{c} VIF=\frac{I\left(C;F\right)}{I\left(C;E\right)}\text{,} \end{array}$$

where *E* is the image perceived by the HVS. *I*(*C*; *F*) and *I*(*C*; *E*) represent the information that the brain can extract in reference and test images, respectively. The larger the VIF value, the better the image fidelity. The results are shown in Table 2.

### 3.3. Subjective Evaluation

**Figure 9:**
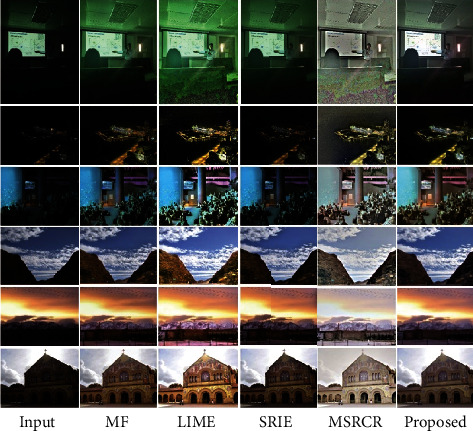
Comparison of the effects of different datasets.

Figure 9 is another dataset on which we tested, and we can see the enhancement effect on low-light images under different methods. From the perspective of human vision, MF, SRIE, and our proposed method can effectively enhance images; the LIME algorithm and the MSRCR method have a poor enhancement effect. This is consistent with the data validation of Tables 3 and 4.

The above enhanced images and data can intuitively show that compared with the current mainstream low-light image enhancement methods, the method proposed in this paper can more effectively reduce the distortion of visual information, and the overall visual effect is better.

## 4. Conclusion

This paper proposes an efficient low-light image enhancement method. The core idea of this method is to synthesize double-exposure images through the camera response model, obtain an optimized light map based on the LIME method, and finally perform postprocessing to further improve the image enhancement effect. The experimental results show that compared with other existing common low-light image enhancement schemes, the method proposed in this paper can achieve better image enhancement effects both subjectively and objectively.

In the future, we should consider environmental factors and avoid over-enhancement due to the unknown environmental conditions. In addition, we can use machine learning-related theories to make further improvements. In our experiments, we used a limited dataset. So, we can try more datasets for more accurate data analysis.

## Figures and Tables

**Table 1:** Comparison of quantitative performance of different methods in terms of LOE.

Method and image	Comparison of quantitative performance of different methods in terms of LOE				
	BOY	SHOE	BOY2	FATHER	BOY3

MSRCR	1600.15	1673.6	1600.33	1940.24	2160.49
SRIE	599.34	618.83	709.31	564.33	499.77
LIME	1388.30	1288.88	1434.57	1368.46	1329.36
MF	742.75	538.23	807.26	652.99	685.92
Proposed	303.49	446.43	289.62	430.12	301.81

**Table 2:** Quantitative performance results of VIF.

Method and image	Quantitative performance results of VIF				
	BOY	SHOE	BOY2	FATHER	BOY3

MSRCR	0.43525	0.29493	0.66658	0.24967	0.47728
SRIE	0.55014	0.50491	0.77618	0.62694	0.72162
LIME	0.30723	0.20269	0.60919	0.31385	0.51933
MF	0.61596	0.45259	0.98047	0.70466	0.84256
Proposed	0.7585	0.68341	0.89024	0.70322	0.8943

**Table 3:** Comparison of quantitative performance of different methods in terms of LOE.

Method	Peng	Dormitory	Screen	Sky	Cloud	Home	LOE(Ave)
MF	275.71	469.04	357.42	642.77	412.88	629.72	464.55
LIME	1382.11	1025.02	1062.62	1175.31	1239.2	1164.51	1174.89
SRIE	283.47	1183.61	412.73	660.49	327.17	701.66	594.88
MSRCR	4442.73	1553.33	1561.13	1797.32	1197.44	1404.82	1992.76
Proposed	525.67	988.44	659.26	405.95	355.22	670.76	600.86

**Table 4:** Comparison of quantization performance of different in terms of VIF.

Method	Peng	Dormitory	Screen	Sky	Cloud	Home	VIF(Ave)
MF	0.62566	0.38453	0.24674	0.62679	0.57326	0.3492	0.4678
LIME	0.19281	0.14978	0.08449	0.33217	0.2367	0.14688	0.19048
SRIE	0.61524	0.54838	0.38424	0.62283	0.55206	0.4555	0.52970
MSRCR	0.16534	0.27782	0.11369	0.45671	0.29517	0.15386	0.24375
Proposed	0.72139	0.63082	0.2164	0.92307	0.87397	0.45882	0.63742

## Data Availability

The data used to support the findings of this study are available from the corresponding author upon request.
